# Supplementary material for: IMPROVING HAND HYGIENE IN CRUISE SHIP: AN INTERVENTION STUDY
Source: Open Res Eur. 2026 Jan 15;5:279. Originally published 2025 Sep 10. [Version 3] doi: 10.12688/openreseurope.20172.3 (PMC12699216; doi:10.12688/openreseurope.20172.3)
Supplement: Peer review history

# Reviewer report: Reviewer response for version 3

**DOI:** 10.21956/openreseurope.24565.r68141

**Published:** 2026-01-21

**Authors:** Colum Dunne

**Affiliations:** 1 University of Limerick, Limerick, County Limerick, Ireland

The authors have dealt with all reviewer comments well.

Is the study design appropriate and does the work have academic merit?

Yes

Is the work clearly and accurately presented and does it cite the current literature?

Partly

If applicable, is the statistical analysis and its interpretation appropriate?

I cannot comment. A qualified statistician is required.

Are all the source data underlying the results available to ensure full reproducibility?

Partly

Are the conclusions drawn adequately supported by the results?

Yes

Are sufficient details of methods and analysis provided to allow replication by others?

Yes

Reviewer Expertise:

Medical microbiology; infection prevention and control; innovation

I confirm that I have read this submission and believe that I have an appropriate level of expertise to confirm that it is of an acceptable scientific standard.

# Reviewer report: Reviewer response for version 3

**DOI:** 10.21956/openreseurope.24565.r68139

**Published:** 2026-01-21

**Authors:** Ida Hellum Sandbekken

**Affiliations:** 1 Oslo Metropolitan University, Oslo, Norway

Thank you for submitting the revised manuscript. All comments raised in the previous review have been adequately addressed, and I am satisfied with the changes made.

 I therefore recommend the manuscript for indexing.

Is the study design appropriate and does the work have academic merit?

Yes

Is the work clearly and accurately presented and does it cite the current literature?

Yes

If applicable, is the statistical analysis and its interpretation appropriate?

Yes

Are all the source data underlying the results available to ensure full reproducibility?

Yes

Are the conclusions drawn adequately supported by the results?

Yes

Are sufficient details of methods and analysis provided to allow replication by others?

Yes

Reviewer Expertise:

Hand hygiene, interventions for improving hand hygiene.

I confirm that I have read this submission and believe that I have an appropriate level of expertise to confirm that it is of an acceptable scientific standard.

# Reviewer report: Reviewer response for version 3

**DOI:** 10.21956/openreseurope.24565.r68140

**Published:** 2026-01-19

**Authors:** Samantha Lange

**Affiliations:** 1 University of Johannesburg, Auckland Park, Gauteng, South Africa

I am happy with version 3 of the article

Is the study design appropriate and does the work have academic merit?

Partly

Is the work clearly and accurately presented and does it cite the current literature?

Partly

If applicable, is the statistical analysis and its interpretation appropriate?

Partly

Are all the source data underlying the results available to ensure full reproducibility?

Partly

Are the conclusions drawn adequately supported by the results?

Partly

Are sufficient details of methods and analysis provided to allow replication by others?

No

Reviewer Expertise:

NA

I confirm that I have read this submission and believe that I have an appropriate level of expertise to confirm that it is of an acceptable scientific standard.

# Reviewer report: Reviewer response for version 2

**DOI:** 10.21956/openreseurope.23773.r64853

**Published:** 2025-12-26

**Authors:** Ida Hellum Sandbekken

**Affiliations:** 1 Oslo Metropolitan University, Oslo, Norway

Thank you for the opportunity to review this interesting paper.

 The relevance is clear, as cruise ships accommodate many people in confined spaces, allowing infections to spread rapidly.

 Overall, this is a well-written paper, but I have some minor suggestions for revision:

1. **Introduction:** I suggest adding a sentence defining hand hygiene, clarifying that it includes both handwashing and the use of disinfectants. Also, ensure there is a clear rationale for when handwashing versus hand disinfection should be used.
2. **Intervention: Surface disinfection:** Regarding surface disinfection, could you add a sentence explaining its relevance to hand hygiene? For example: *Frequently touched surfaces play a significant role in infection transmission because…*
3. **Intervention: Hand Hygiene Training:** The first two sentences are unclear. You refer to “small groups” twice: first, *Crew attended in small groups*, then *Crew’s hand hygiene training was conducted in small groups.* Is this repetition, meaning the second phrase can be deleted? Or are these two different groupings?
4. **Outcomes: Hand Hygiene Compliance Rate:** You use the term *handwashing* (Please check your manuscript for consistency in using “handwashing” (one word) rather than “hand washing”). However, I think you may be referring to hand disinfection instead—please clarify.
5. **Outcomes: Questionnaire: **Why did some participants complete the questionnaire before and others after the training? Were they the same individuals? Additionally, I suggest noting that participation was voluntary.
6. The heading **“The Cruise Ship”** currently appears under the bold heading **“Outcomes.”** Could it be moved to a more appropriate section?
7. **Results: Environmental Microbiological Samples (page 9):** Can the figures be placed in the correct order? Currently, Figure 8B appears before 8A. You may need to rename the figures if you want to maintain the sentence order.
8. **Interventions: Technology-Induced Behavioral Change (page 15):** Please correct the grammar in this sentence: *The researchers asked **the** some of the crew why they were not using the system more frequently.*
9. **Interventions: Hand Hygiene Training:** The sentence *“In all four questions, there was some shift, with more participants answering correctly after the training than before”* is unclear. Which four questions? Also, the word *shift* is vague, consider using a more descriptive term.
10. **Discussion:** The phrase *“A single traumatic event led to a 15%…”, *are you referring to 84.5 – 60.4 = 24.1? Where does 15% come from?
11. In the second-to-last paragraph, *“The hand hygiene experiment…”, *are you referring to the Semmelweis hand hygiene system? If so, use the same term; if not, clarify what you mean.

Is the study design appropriate and does the work have academic merit?

Yes

Is the work clearly and accurately presented and does it cite the current literature?

Yes

If applicable, is the statistical analysis and its interpretation appropriate?

Yes

Are all the source data underlying the results available to ensure full reproducibility?

Yes

Are the conclusions drawn adequately supported by the results?

Yes

Are sufficient details of methods and analysis provided to allow replication by others?

Yes

Reviewer Expertise:

Hand hygiene, interventions for improving hand hygiene.

I confirm that I have read this submission and believe that I have an appropriate level of expertise to confirm that it is of an acceptable scientific standard, however I have significant reservations, as outlined above.

# Response

**Published:** 2026-01-08

**Authors:** Jörn Klein

**Affiliations:** Faculty of Technology, Natural Sciences and Mariti, Universitetet i Sorost-Norge, Porsgrunn, Telemark, Norway

We are grateful to the reviewers for their careful assessment of our manuscript and for the insightful and constructive comments. We appreciate the time and effort invested in the review process. The suggestions provided were highly valuable and helped us improve the clarity of the manuscript, refine the presentation of the methods and results, and enhance the overall quality of the work. Below, we address each comment in detail and describe how the manuscript was revised in response.

 Please note that some references were renumbered following the relocation of the WHO reference from [9] to [5].”  

**Reviewer#4 - Ida Hellum Sandbekken**

 Thank you for the opportunity to review this interesting paper. The relevance is clear, as cruise ships accommodate many people in confined spaces, allowing infections to spread rapidly. Overall, this is a well-written paper, but I have some minor suggestions for revision:

**1.** Introduction: I suggest adding a sentence defining hand hygiene, clarifying that it includes both handwashing and the use of disinfectants. Also, ensure there is a clear rationale for when handwashing versus hand disinfection should be used.

**Response: **The following sentences was added to the Introduction section: P4 L54: “Proper hand hygiene is the first line of defense against the spread of many illnesses on board [4]. Hand hygiene is a general term referring all actions of hand cleansing, including handwashing with soap and water and hand rubbing with alcohol-based hand rubs. Hand hygiene recommendations provide detailed guidance on when hand rubbing or handwashing should be applied [5].”  

**2.** Intervention: Surface disinfection: Regarding surface disinfection, could you add a sentence explaining its relevance to hand hygiene? For example: Frequently touched surfaces play a significant role in infection transmission because…

**Response: **Thanks for pointing out: P7 L100: “Frequently touched surfaces are significant in infection transmission, as microorganisms may persist on surfaces and recontaminate hands during contact, increasing the risk of cross-transmission. Decontamination of these surfaces minimizes environmental sources of hand contamination [5].”  

**3**. Intervention: Hand Hygiene Training: The first two sentences are unclear. You refer to “small groups” twice: first, Crew attended in small groups, then Crew’s hand hygiene training was conducted in small groups. Is this repetition, meaning the second phrase can be deleted? Or are these two different groupings?

**Response: **These were the same, the section was rephrased: P9 L141: “Hand hygiene training sessions were conducted by USN researchers on October 30th, 31st, and November 2nd. Crew members attended in small groups of approximately 15 people each. The training sessions lasted between 20 and 25 minutes. The crew’s hand hygiene training was conducted in small groups (approximately 15 people each) and structured as an interactive discussion rather than a traditional lecture.”  

**4.** Outcomes: Hand Hygiene Compliance Rate: You use the term handwashing (Please check your manuscript for consistency in using “handwashing” (one word) rather than “hand washing”). However, I think you may be referring to hand disinfection instead—please clarify. **Response: **P12 L198: “Three key moments where selected where hand hygiene would be necessary and observation was feasible” Handwashing (one word) was corrected in L569, L571 and L574.  

**5**. Outcomes: Questionnaire: Why did some participants complete the questionnaire before and others after the training? Were they the same individuals? Additionally, I suggest noting that participation was voluntary.

**Response: **The original plan was for all participants to complete the questionnaire before the training, but for logistical reasons, half of the participants received the invitation only after the training. This provided an opportunity to compare results before and after the training. Each participant completed the questionnaire only once, either before or after the training. Explanation was added: P28 L453: “A total of 179 crew members completed the online questionnaire: 69 before the training and 89 after (each participant completed the questionnaire only once, either before or after the meeting, depending on their schedule).” The following sentence was added: P14, L227: “Crew members were asked to fill out the questionnaire through their supervisors. Participation was entirely voluntary.”  

**6. **The heading “The Cruise Ship” currently appears under the bold heading “Outcomes.” Could it be moved to a more appropriate section?

**Response: **Right, it was moved. Now it started in P6, L89  

**7.** Results: Environmental Microbiological Samples (page 9): Can the figures be placed in the correct order? Currently, Figure 8B appears before 8A. You may need to rename the figures if you want to maintain the sentence order.

**Response: **Figure 8 was changed: refer following link - https://openreseurope-files.f1000.com/linked/266544.Ms_id_20172-_Fig.8.jpg 

 P22 L344: “Passengers had the opportunity to watch the daily show at the Muses Lounge, enjoy drinks at the bar, or dance at the Selene Lounge (Figure 8A). As shown in the traffic data, there was a continuous, elevated flow of people for several hours during these events (Figure 8B).” P22 L353: “Figure 8: Traffic data and sampling sites during late-night shows. Sample collection sites are marked with a red X on the floor plan. The figure shows the number of people who exited Deck 5 using the left midship staircase (downward).”  

**8**. Interventions: Technology-Induced Behavioral Change (page 15): Please correct the grammar in this sentence: The researchers asked the some of the crew why they were not using the system more frequently.

**Response: **Corrected: P32 L518: “The researchers asked some of the crew why they were not using the system more frequently.”  

**9**. Interventions: Hand Hygiene Training: The sentence “In all four questions, there was some shift, with more participants answering correctly after the training than before” is unclear. Which four questions? Also, the word shift is vague, consider using a more descriptive term. **Response: **Rephrased: P34 L559: “In all four questions presented in Figure 16, a higher proportion of participants answered correctly after the training than before (correct answers are marked with a star in Figure 16); however, the changes were statistically significant in only two cases (Table 5). We were not fully satisfied with the extent of this improvement, indicating that there is still room for improvement how the message was targeted.”  

**10**. Discussion: The phrase “A single traumatic event led to a 15%…”, are you referring to 84.5 – 60.4 = 24.1? Where does 15% come from?

**Response: **Thanks for noticing it! Yest, it should be 25% P38 L634: “A single traumatic event led to an approximately 25% drop in compliance (from 84.5% to 60.4%, Table 4) in the following weeks, potentially increasing the infection risk for thousands of passengers.”  

**11. **In the second-to-last paragraph, “The hand hygiene experiment…”, are you referring to the Semmelweis hand hygiene system? If so, use the same term; if not, clarify what you mean. **Response:** No, we tried to refer to the fingerprint-culture experiment. P39, L653: “The hand hygiene experiment (Figure 2, performed during crew training) was especially effective, as it allowed participants to witness the results firsthand rather than simply being told what to believe.”

# Reviewer report: Reviewer response for version 2

**DOI:** 10.21956/openreseurope.23773.r64632

**Published:** 2025-12-11

**Authors:** Samantha Lange

**Affiliations:** 1 University of Johannesburg, Auckland Park, Gauteng, South Africa

Thank you for addressing the comments from my previous review. As you stated in your rebuttal, this study should be viewed as a pilot study. I would recommend that you bring this in to the manuscript as this will provide a more transparent view of the study and explain some of the concerns that I had and that future readers may have. I would also suggest that you bring in recommendations for future studies such as using a validated tool, expanding the sample size to include more ships etc. The information will be very valuable in a space where little information is available.

Is the study design appropriate and does the work have academic merit?

Partly

Is the work clearly and accurately presented and does it cite the current literature?

Partly

If applicable, is the statistical analysis and its interpretation appropriate?

Partly

Are all the source data underlying the results available to ensure full reproducibility?

Partly

Are the conclusions drawn adequately supported by the results?

Partly

Are sufficient details of methods and analysis provided to allow replication by others?

No

Reviewer Expertise:

D Tech Environmental Health with published research in hand hygiene interventions as well as 30 years experience in the environmental health/ food hygiene/hygiene foeld

I confirm that I have read this submission and believe that I have an appropriate level of expertise to confirm that it is of an acceptable scientific standard, however I have significant reservations, as outlined above.

# Response

**Published:** 2026-01-08

**Authors:** Jörn Klein

**Affiliations:** Faculty of Technology, Natural Sciences and Mariti, Universitetet i Sorost-Norge, Porsgrunn, Telemark, Norway

Thank you for addressing the comments from my previous review. As you stated in your rebuttal, this study should be viewed as a pilot study. I would recommend that you bring this in to the manuscript as this will provide a more transparent view of the study and explain some of the concerns that I had and that future readers may have. P40 L661: “Our paper is rather a report, and not a standardized, large-scale study. Hand hygiene research and education on cruise ships is an underinvestigated area. Most of the methods we used were originally developed for hospital environments. For example, hand hygiene technique monitoring has never been implemented on any cruise ship before. As our paper highlights, not all of the solutions that work in hospitals can be directly applied to such different settings. We believe that our work provides several new insights that can help other professionals further develop and adapt these methods for use in the cruise ship industry”.   I would also suggest that you bring in recommendations for future studies such as using a validated tool, expanding the sample size to include more ships etc. The information will be very valuable in a space where little information is available. The following paragraph was added: P43, L726: “Recommendations for future studies: During compliance measurements, we observed that factors such as age, sex, and nationality may influence hand hygiene compliance; future studies should specifically address these parameters. We also found that strong management commitment is necessary to successfully implement new interventions. Crew members are often busy and fatigued, and infection control is not a priority for them; as a result, non-mandatory tasks are frequently overlooked. Hand hygiene training should be tailored to the target audience, taking into account that many crew members have limited formal education in biology and that language barriers may exist. Training should begin with basic concepts and rely heavily on visual materials, such as pictures, videos, and diagrams, rather than text.”

# Response

**Published:** 2026-01-16

**Authors:** Samantha Lange

**Affiliations:** Not Applicable, South Africa

Thank you, my previous concerns have been adequately addressed.

# Reviewer report: Reviewer response for version 2

**DOI:** 10.21956/openreseurope.23773.r64852

**Published:** 2025-12-11

**Authors:** Sudip Bhattacharya

**Affiliations:** 1 All India Institute of Medical Sciences, Deoghar, Jharkhand, India

Major Revision

**Title & Abstract**

- The title does not specify the study design clearly.
- Abstract is descriptive but lacks concise presentation of numerical outcomes; background and results sections read too narratively.

**Introduction**

- Some claims are overly general and not specific to cruise-ship epidemiology.
- Literature cited is insufficiently integrated to justify the multimodal intervention approach.

**Methods**

- Study design relies on sequential arms without a parallel control, which limits causal inference.
- Sampling strategy for environmental microbiology (only eight surfaces) is narrow and not fully justified.
- Questionnaire development process is not described in detail; validity and reliability remain unclear.
- The description of intervention components is lengthy and could be streamlined for clarity.

**Results**

- Several key results (e.g., CFU counts, compliance percentages) are repeated in multiple sections, reducing clarity.
- The hand hygiene compliance datasets may include repeated observations of the same individuals, but this limitation appears late rather than upfront.
- Interpretation of cultural and behavioral observations is anecdotal and lacks systematic measurement.
- Visual elements (Figures 6–12) require clearer captions to improve interpretability.

 
**Discussion**

- Discussion reiterates findings already stated in the Results section, creating redundancy.
- Some behavioural interpretations (e.g., passenger excuses, cultural differences) are speculative and not supported by structured qualitative data.
- The narrative is long and would benefit from sharper thematic organisation.
- Comparison with international literature is limited and should be expanded to contextualise why interventions failed.

 
**Limitations**

- Although several limitations are acknowledged, the section is verbose and includes methodological clarifications that should appear earlier.
- Observer bias (Hawthorne effect) and selection bias among crew respondents should be addressed more precisely.

**Conclusion**

- The conclusion is lengthy and introduces new information (e.g., passenger behaviour categories) instead of synthesising the study’s findings.
- Recommendations remain broad and require clearer alignment with the study results.

**Language & Formatting**

- Multiple grammatical errors, repetitive phrasing, and inconsistent use of terminology appear throughout the manuscript.
- Figures and tables require more consistent formatting and clearer explanatory notes.

Additionally, the authors must add behavioural aspects, nudge theories and use of AI to improve the manuscript.

https://pubmed.ncbi.nlm.nih.gov/39917534/

https://icjournal.org/DOIx.php?id=10.3947/ic.2024.0147

https://journals.lww.com/chri/fulltext/2019/06010/namastey___greet_the_indian_way__reduce_the_chance.17.aspx

Is the study design appropriate and does the work have academic merit?

Yes

Is the work clearly and accurately presented and does it cite the current literature?

Yes

If applicable, is the statistical analysis and its interpretation appropriate?

Yes

Are all the source data underlying the results available to ensure full reproducibility?

Yes

Are the conclusions drawn adequately supported by the results?

Yes

Are sufficient details of methods and analysis provided to allow replication by others?

Yes

Reviewer Expertise:

Public Health

I confirm that I have read this submission and believe that I have an appropriate level of expertise to confirm that it is of an acceptable scientific standard, however I have significant reservations, as outlined above.

# Reviewer report: Reviewer response for version 2

**DOI:** 10.21956/openreseurope.23773.r64631

**Published:** 2025-11-24

**Authors:** Colum Dunne

**Affiliations:** 1 University of Limerick, Limerick, County Limerick, Ireland

Thank you for the opportunity to read this revised manuscript. 

 The authors have made a considerable number of revisions and, in particular, have detailed the limitations of the study more explicitly. This is an important amendment and clarifies the rationale, methods and outcomes of the study to a much great degree.

 In turn, this allows any reader to judge the study and its relevant to them much easier and with a greater level of insight.

 Overall then, this is a significant improvement on the first draft.

Is the study design appropriate and does the work have academic merit?

Yes

Is the work clearly and accurately presented and does it cite the current literature?

Partly

If applicable, is the statistical analysis and its interpretation appropriate?

I cannot comment. A qualified statistician is required.

Are all the source data underlying the results available to ensure full reproducibility?

Partly

Are the conclusions drawn adequately supported by the results?

Yes

Are sufficient details of methods and analysis provided to allow replication by others?

Yes

Reviewer Expertise:

Medical microbiology; infection prevention and control; innovation

I confirm that I have read this submission and believe that I have an appropriate level of expertise to confirm that it is of an acceptable scientific standard.

# Reviewer report: Reviewer response for version 1

**DOI:** 10.21956/openreseurope.21824.r62861

**Published:** 2025-11-06

**Authors:** Colum Dunne

**Affiliations:** 1 University of Limerick, Limerick, County Limerick, Ireland

Thank you for the invitation to review this manuscript. The study is interesting and reports hand hygiene practices in a cruise ship setting.

 There are many limitations to the study, including but not limited to its single performance and the demographics of passengers who might be able to travel in such a way based on their education/professional or socio-economic attainment. The authors have dealt with this to some degree but this could be expanded upon.

 I was very interested in the study as it is a variation of studies performed in education of clinical settings. I think it would be of interest to the actors and readers to access these two papers that report the varying characteristics, successes and challenges of hand hygiene studies internationally and for the authors to then address similarities and difference as observed in their report.

 The studies are: https://www.sciencedirect.com/science/article/abs/pii/S0195670115004892 (based on studies published between 2010 and 2015) 

 and

 https://www.sciencedirect.com/science/article/pii/S019567012100102X (based on studies published between 2014 and 2020)

Is the study design appropriate and does the work have academic merit?

Yes

Is the work clearly and accurately presented and does it cite the current literature?

Partly

If applicable, is the statistical analysis and its interpretation appropriate?

I cannot comment. A qualified statistician is required.

Are all the source data underlying the results available to ensure full reproducibility?

Partly

Are the conclusions drawn adequately supported by the results?

Yes

Are sufficient details of methods and analysis provided to allow replication by others?

Yes

Reviewer Expertise:

Medical microbiology; infection prevention and control; innovation

I confirm that I have read this submission and believe that I have an appropriate level of expertise to confirm that it is of an acceptable scientific standard, however I have significant reservations, as outlined above.

# Response

**Published:** 2025-11-18

**Authors:** Jörn Klein

**Affiliations:** Faculty of Technology, Natural Sciences and Mariti, Universitetet i Sorost-Norge, Porsgrunn, Telemark, Norway

We would like to sincerely thank the reviewers for their thorough evaluation of our manuscript and for the constructive comments provided. We greatly appreciate the time and effort dedicated to reviewing our work. The feedback has been extremely valuable in helping us clarify key points, improve the presentation of our methods and results, and strengthen the overall quality of the manuscript. Below, we provide a detailed point-by-point response to each comment, indicating how the manuscript has been revised in response to the suggestions. Please note that three additional references have been added, resulting in the renumbering of the reference list. Additionally, since Figure 15 was moved earlier in the manuscript, the numbering of nearly all figures has also changed.  

**Reviewer#2 - Colum Dunne**   Thank you for the invitation to review this manuscript. The study is interesting and reports hand hygiene practices in a cruise ship setting. There are many limitations to the study, including but not limited to its single performance and the demographics of passengers who might be able to travel in such a way based on their education/professional or socio-economic attainment. The authors have dealt with this to some degree but this could be expanded upon. I was very interested in the study as it is a variation of studies performed in education of clinical settings. I think it would be of interest to the actors and readers to access these two papers that report the varying characteristics, successes and challenges of hand hygiene studies internationally and for the authors to then address similarities and difference as observed in their report. The studies are: https://www.sciencedirect.com/science/article/abs/pii/S0195670115004892 (based on studies published between 2010 and 2015) and https://www.sciencedirect.com/science/article/pii/S019567012100102X (based on studies published between 2014 and 2020)   Thank you for reviewing the manuscript.

 You are clear, the purpose of the study was to explore how hand hygiene interventions that are effective in clinical settings can be applied in a different context. The two articles you suggested is very valuable, thank you for pointing them out. We have added both as references, and we incorporated the following text into the manuscript: L61 “In healthcare settings, hand hygiene interventions have been more extensively researched and documented. Most of these studies use multimodal approaches, combining a variety of strategies aimed at addressing barriers to improving compliance. Multimodal strategies almost always include some form of education or training, as well as compliance monitoring [5]. However, another review concluded that both multimodal and single-intervention studies can achieve modest to moderate improvements in hand hygiene compliance [6]. Our study implemented a multimodal strategy to improve hand hygiene, incorporating antimicrobial surface treatments, a technology-based hand hygiene performance monitoring, and targeted training. These elements were introduced stepwise, each added on top of the previous one.”

# Reviewer report: Reviewer response for version 1

**DOI:** 10.21956/openreseurope.21824.r60603

**Published:** 2025-10-06

**Authors:** Samantha Lange

**Affiliations:** 1 University of Johannesburg, Auckland Park, Gauteng, South Africa

This manuscript will benefit from major revisions. The methodology does not describe all aspects of the study that are discussed later in the paper. The results do not show any analytical results even though in some cases there was mention made of this. The discussion does not compare results to other studies. The researchers make use of many of their own observations and thoughts while not quantifying these in any way. Many of the limitations are discussed as outcomes. Outcomes should fall under the results section. The conclusions need to be directly linked to the results. In general this reads as a report and not as scientific, replicable, relevant research. My comments have been inserted onto the manuscript: *https://openreseurope-files.f1000.com/linked/259660.813bdc0e_fc49_4679_aaa1_a749c2f258d6_20172___jorn_klein_comments.pdf*

Is the study design appropriate and does the work have academic merit?

Partly

Is the work clearly and accurately presented and does it cite the current literature?

Partly

If applicable, is the statistical analysis and its interpretation appropriate?

Partly

Are all the source data underlying the results available to ensure full reproducibility?

Partly

Are the conclusions drawn adequately supported by the results?

Partly

Are sufficient details of methods and analysis provided to allow replication by others?

No

Reviewer Expertise:

D Tech Environmental Health with published research in hand hygiene interventions as well as 30 years experience in the environmental health/ food hygiene/hygiene foeld

I confirm that I have read this submission and believe that I have an appropriate level of expertise to state that I do not consider it to be of an acceptable scientific standard, for reasons outlined above.

# Response

**Published:** 2025-11-18

**Authors:** Jörn Klein

**Affiliations:** Faculty of Technology, Natural Sciences and Mariti, Universitetet i Sorost-Norge, Porsgrunn, Telemark, Norway

We would like to sincerely thank the reviewers for their thorough evaluation of our manuscript and for the constructive comments provided. We greatly appreciate the time and effort dedicated to reviewing our work. The feedback has been extremely valuable in helping us clarify key points, improve the presentation of our methods and results, and strengthen the overall quality of the manuscript. Below, we provide a detailed point-by-point response to each comment, indicating how the manuscript has been revised in response to the suggestions. Please note that three additional references have been added, resulting in the renumbering of the reference list. Additionally, since Figure 15 was moved earlier in the manuscript, the numbering of nearly all figures has also changed. 

**Comment:**

 This manuscript will benefit from major revisions. The methodology does not describe all aspects of the study that are discussed later in the paper. The results do not show any analytical results even though in some cases there was mention made of this. The discussion does not compare results to other studies. The researchers make use of many of their own observations and thoughts while not quantifying these in any way. Many of the limitations are discussed as outcomes. Outcomes should fall under the results section. The conclusions need to be directly linked to the results. In general, this reads as a report and not as scientific, replicable, relevant research. My comments have been inserted onto the manuscript: https://openreseurope-files.f1000.com/linked/259660.813bdc0e_fc49_4679_aaa1_a749c2f258d6_20172___jorn_klein_comments.pdf

**Response: **The authors acknowledge that our paper is not a standardized, replicable, large-scale study; rather a report. Hand hygiene research and education on cruise ships is an under investigated area. Most of the methods we used were originally developed for hospital environments. For example, hand hygiene technique monitoring has never been implemented on any cruise ship before. As our paper highlights, not all of the solutions that work in hospitals can be directly applied to such different settings. Although our work is more of a report, we believe it provides several new insights that can help other professionals further develop and adapt these methods for use in the cruise ship industry. Ultimately, this may contribute to standardized, large-scale data collection in the future. However, this development can only progress if our report is published, as publication is the primary way knowledge is shared within the scientific community.   Quantify some of your significant results here The following additional result was included: L19: “Staff members missed an average of 9.6% of hand surface during hand rubbing when hand hygiene was first monitored.” Also to the Results / Technology induced behavioral change section: L495: “The Semmelweis System was installed onboard on September 1, 2023, and remained operational until November 9. During this period, a total of 1’636 measurements were conducted, with 204 QR codes in use. Analyzing only the first measurements of the participants, an average of 9.6% of the hand surface was not covered by hand rub.”   Your conclusion doesn't speak to your objectives and study results The following sentences were added: L25: “Conclusions Surface disinfection, technique monitoring, and passenger training did not improve hand hygiene in the forms applied in this study. Crew training improved theoretical knowledge, but we were not able to demonstrate any measurable behavioural change. There were some additional lessons from the study.”   Where these passengers or crew? Where there inclusions or exclusions? Both passengers and crew members were included, depending on the specific intervention. This is described in the sections detailing each intervention. There were no additional inclusion criteria beyond being on board: L82. “All participants were recruited exclusively onboard the Celestyal Olympia, with no other sites involved. No exclusion criteria were applied.”   Why did you choose these two sprays? As mentioned later in the manuscript (L631), we tested four sprays in a previous study that is unfortunately still under review. The antibacterial performance of the four sprays was similar. However, one spray consisted of two components, and another left a grey residue on surfaces. Therefore, we selected the remaining two sprays for the current study.   Did you follow manufacturers instructions for application regarding frequency, re-application, preparation of surface prior to application? Yes, we followed the manufacturer’s instructions during application. Re-application was not performed, as one spray claims a 30-day effect and the other a 60-day effect (L102). Sampling took place 1–2 days after the sprays were applied. L106: “The manufacturer’s instructions were followed precisely during application. For Arm2, sampling was conducted 1–2 days after the spray treatment.”   How were hands disinfected and when? Hands were disinfected immediately before using the hand hygiene monitoring device. Participants were informed that the purpose of the measurement was to assess whether they were able to cover the entire surface of their hands with hand rub. No additional instructions were provided regarding the disinfection technique.   How was the training material validated? As it was mentioned in the Result section, the content was continuously refined based on feedback collected by the researcher. This description was relocated form the Result section to the Methods. L130: “Hand hygiene training sessions were conducted by USN researchers on October 30th, 31st, and November 2nd. Crew members attended in small groups of approximately 15 people each. The training sessions lasted between 20 and 25 minutes. The crew’s hand hygiene training was conducted in small groups and structured as an interactive discussion rather than a traditional lecture. The content was continuously refined based on feedback collected by the researcher. Over the course of the three days, a total of 367 crew members participated.” The training was not formally validated. The latest version has been published to serve as the basis for a future, validated training program.   Was this flyer piloted prior to use? How was information and wording validated? The flyer was not piloted prior to use. The study itself should be considered a pilot.   How did you determine frequently touched surfaces and sample frequency? Frequently touched surfaces were selected by the researcher onboard. Many studies have focused on microbial loads on frequently touched surfaces in hospitals [https://pmc.ncbi.nlm.nih.gov/articles/PMC7087772/, https://www.cdc.gov/healthcare-associated-infections/hcp/cleaning-global/appendix-c.html]. However, many of these surfaces are not found on cruise ships. The researcher observed passenger behavior during her first days onboard, to identify the most frequently touched surfaces. To verify these observations, touch frequency was measured and is presented in Figures 6, 7, and 8.   Why were these determined to be critical moments? Is there literature to support this? Are there HW facilites at these points to assist with HW? We used the “Advice to International Travellers for a Healthy Voyage,” published by the EU Shipsan Act Joint Action, as a starting point. This document provides a list of situations in which hand hygiene is necessary.

- Before and after eating
- Before touching your eyes, nose or mouth
- After coughing, sneezing, or blowing your nose
- Before, during, and after preparing food (make sure you also wash in between handling meat or fish and moving on to other food items)
- After using the restroom
- After changing a diaper
- After touching plants or soil
- After visiting the ships’ hospital
- After coming into contact with any body fluids or touching items that may have come in contact with body fluids (i.e. runny nose, watery eyes, saliva, blood, urine)
- After touching pets or other animals
- After touching surfaces such as door knobs or railings, which are subject to high hand contact.
- After returning to your cabin

Some items on the Shipsan list were not relevant for passengers on board. For example, there were no pets of plants on the ship, and all passengers were on a full-board plan with four meals provided daily, so no one prepared food. These irrelevant moments are labeled in blue. Some moments could not be observed for privacy reasons and are labeled in green. Other moments occurred too rarely to be reliably observed on a large scale by a single observer; these are labeled in orange.

- Before and after eating
- Before touching your eyes, nose or mouth
- After coughing, sneezing, or blowing your nose
- Before, during, and after preparing food (make sure you also wash in between handling meat or fish and moving on to other food items)
- After using the restroom
- After changing a diaper
- After touching plants or soil
- After visiting the ships’ hospital
- After coming into contact with any body fluids or touching items that may have come in contact with body fluids (i.e. runny nose, watery eyes, saliva, blood, urine)
- After touching pets or other animals
- After touching surfaces such as door knobs or railings, which are subject to high hand contact.
- After returning to your cabin

This left us with the moments before and after eating, and after touching surfaces. We also considered the WHO hospital hand hygiene guideline, the “5 Moments for Hand Hygiene.”

*https://openreseurope-files.f1000.com/linked/262826.Image_1.png*

 Moments 1 and 4 (before and after touching a patient) are often operationalized in hospitals as before and after entering a patient’s room, ensuring that nothing is brought into or taken out of the room via the hands. Based on these considerations, we selected the following three moments for observation, where hand hygiene compliance could be reliably measured.

*https://openreseurope-files.f1000.com/linked/262827.image_2*

 How did you control for bias during observation? Were observers trained? All measurements were conducted by a single observer, so inter-observer variability was not a factor. Although the observer did not receive specific training for this study, she is a researcher with over 10 years of experience in hand hygiene research. L196: “We recorded the number of people who performed hand hygiene and those who did not. All measurements were conducted by a single observer, so inter-observer variability was not a factor.”   Were the passengers exposed to the other interventions or were the other interventions only directed to crew members? This is not clear in your methodolgy. The cruise company requested that passengers not be disturbed during their vacation, which typically lasted only 3–4 days. As a result, passengers were only involved in observation-type measurements (e.g., whether or not they performed hand hygiene) and were not asked to take any actions such as completing questionnaires or participating in training. Crew members were excluded from the compliance measurements because, after a few days, they became aware of the sole observer’s identity and subsequently adjusted their behavior; performing hand hygiene whenever they encountered the researcher. For this reason, the study used a mixed-methods approach.   Open or closed ended questions? Binary or Likert-scale? Explain the questionnaire Supplementary Table 2 contains the full questionnaire. (L219, L745)   How was this questionnaire validated? The questionnaire was not validated. The study itself should be considered a pilot.   Why was this not done pre- and post-intervention for all? Each crew member completed the questionnaire once. The link was provided to all participants during their team meeting session. Responses were collected between October 30 and November 2. Meanwhile, training sessions were organized in small groups of approximately 15 people each, held also between October 30 and November 2. This means that some participants completed the questionnaire before the training, while others completed it afterward, depending on their shift. It was challenging to motivate the crew to take the time to complete the questionnaire. We are confident that if they had been asked to complete it twice, even fewer participants would have done so.   How did you compare? Did the same crew members complete before and after? In your previous paprgraph you stated only some completed before and some after. This is confusing A total of 179 crew members completed the online questionnaire: 69 before the training and 89 after. The remaining respondents did not answer this question. Because assignment to pre- or post-training groups was random and determined solely by shift schedules, we assumed that hand hygiene–related knowledge was similar between groups before the training. Therefore, any differences observed between the pre- and post-training groups can reasonably be attributed to the hand hygiene training itself. L450: “A total of 179 crew members completed the online questionnaire: 69 before the training and 89 after. The remaining respondents did not answer this question.”   How did you control for possible intimidation or power-plays if supervisors provided the information? We did not formally control for this. The questionnaire was anonymous and administered online, focusing solely on hand hygiene. Only the researcher had access to the individual responses; supervisors and management received only aggregated, analyzed data. Supervisors simply asked crew members to complete the questionnaire and provided as much time as was feasible under the circumstances. Participation was clearly voluntary, as evidenced by the fact that 179 out of approximately 600 crew members chose to complete the questionnaire.   What about the passengers? As noted earlier, passengers participated only in observations in which we recorded whether hand hygiene was performed in specific situations. No personal data were collected, and no information that could identify individual passengers was recorded. Consequently, the ethics committee determined that individual informed consent was not necessary.   Indicate p values in the table (Figure 8) Modified

*https://openreseurope-files.f1000.com/linked/262828.image_3.jfif*

 This should be presented as a limitation We have added this to the Limitations section, although we believe it should also be mentioned here. L689: “Individual passengers could not be identified; therefore, some may have been observed more than once.”   There is no mention of s seperate measurement for buffet in the methodology. Also two different approaches were applied here. The intervention should be consistent at each HH point. We did not initially plan to measure the Buffet and the Restaurant separately. During the observations we noticed a substantial difference between the two settings, which is why the data are presented separately. The difference appears to be related to the two different approaches applied in these areas. It is important to emphasize that we were not aware of this difference prior to the study. These practices were not part of our intervention and remained consistent across all four arms. Our intended intervention to improve passenger compliance was the distribution of flyers.   How many? Was this significant? We did not record how many passengers actively refused hand rub or how many missed hand hygiene for logistical reasons. It was not feasible to document the reason of non-compliance- The observer watched from a distance of 3–5 meters, conversations were only noticeable during quieter periods. Nevertheless, since these active refusals happened repeatedly, we believe it is important to mention.   You cannot make inferences based on your personal opinion. Please remove this We believe it is important to understand why people do not comply with hand hygiene recommendations. Identifying barriers is essential in order to address them. During our observations, we noted several such barriers. The reason “I just had a shower” may initially seem illogical, hence we attempted to describe the broader context in which it occurred. These observations are not presented as facts but rather as insights that may help guide future researchers to explore these aspects further.   How was this information captured and analysed? This was not in the methodology The researcher observed complaints about the gel’s stickiness repeatedly during compliance observations and in everyday situations on board, such as while waiting in line to enter the restaurant. Crew members also reported during training sessions that passengers frequently complained about the handrub and noted that they themselves did not like it. Some noted that other gels are less sticky and that they would use handrub more frequently if a different product were provided. Even the Provision Master mentioned that they had considered switching to another gel due to passenger complaints. Unfortunately, the issue is more complex. All hygiene products on board (soap, shampoo, lotion, etc.) are supplied by the same provider. For a cruise ship, procurement is a significant logistical challenge, and the company concluded that it is safer to rely on a single trusted supplier capable of delivering all hygiene products in coordinated shipments, rather than switching to a different gel and potentially jeopardizing the supply chain.   Why was this handrub chosen if this was the result? We did not select the handrub. The same product that had been used previously remained in use throughout the study.   You can't have an impression- provide results to back up this statement We did not make recordings during the study, so retrospective analysis is not feasible. The single researcher observed more than 22000 hand hygiene opportunity, and we believe this provided a sufficient basis for forming an impression. Our study was not designed to address age-related differences, as we had no prior indication that age might be an important factor. For this reason, we consider it valuable to mention this topic, as it may help future researchers explore the issue more systematically and generate data that could either support or refute our impression.   Quantify these results and state whether this is significant We cannot quantify this observation; it is an impression that we report solely to inform other researchers and to support the design of future studies that may focus on cultural differences in hand hygiene related to nationality. Many passengers traveled in groups with designated group leaders, which made their nationality recognizable.   This is a limitation Our study demonstrated that the location of the dispensers had a substantial impact on hand hygiene compliance. The purpose of the study was to identify ways to improve hand hygiene on a cruise ship, and although many of our interventions did not show significant effects, optimizing dispenser placement clearly emerged as a strategy that can meaningfully improve compliance. Additional details are provided in our abstract: https://drive.google.com/file/d/1FKYe0nOd8wO5aZTt1Pdi3YF9gAwgXiHq/view?usp=sharing   Figure 8 does not talk to a pillow letter's effectiveness- explain in more detail here Thank you for the correction, it is Figure 10 (former Figure 9). L525: “The passengers' pillow letter was ineffective (Figure 9).”   Another limitation We believe it is more an explanation of the experimental data.   This should form part of your methodology Relocated, as well as Figure 15.   Was this the same crew members or different ones? If so, why? As I was mentioned above, 69 crew members completed the online questionnaire before the training and 89 after. L450: “A total of 179 crew members completed the online questionnaire: 69 before the training and 89 after. The remaining respondents did not answer this question.”   Provide p values for this statement. p values were added to the relevant figure (Figure 8). P values were also added to the Results part: L352: “There was no statistically significant reduction in bacterial counts on treated surfaces in Arm 2 compared to baseline (p = 0.368). Similarly, neither Arm 3 nor Arm 4 showed significant differences in surface CFUs from baseline (p = 0.673 and 0.251, respectively).”   Link to other studies reference added L606: “The location of the dispensers largely influenced hand hygiene compliance [18].”   How can you be sure this is the reason. Has something similar been proven to be the reason in previous studies? At the restaurant entrance, hand hygiene compliance was consistently high, mainly because the hostess actively offered handrub to everyone. It was observed that whenever the hostess temporarily left her position (for example, when a large group arrived and she stepped inside to check whether seating was available), compliance dropped immediately. During Arm4, the data showed a decrease in compliance, and the researcher noticed that this was because the hostess was less proactive in providing handrub. Previously, she asked every passenger to perform hand hygiene. During Arm4, she merely held up the spray bottle and dispensed it only if passengers extended their hands. This was a substantial difference, clearly observed by the researcher. During quieter periods, the researcher often had conversations with the hostess, as both were worked in the same place for weeks. In one of these conversations, the hostess shared the incident that we report. Because the researcher considered it important, she asked the hostess for permission to include this information in the study.   How did you encounter these? Interviews with crew was not part of your methodology. If this is a result it should be in the results section There were no formal interviews with the crew. The researcher spent seven weeks on board and had both formal, planned interactions with the staff (training) as well as informal, unplanned ones. Crew members quickly noticed that the researcher presence had no negative consequences; for example, when she observed something, no manager arrived to demand corrections. Crew members also learned many personal details about the researcher, such as when she woke up, the color of her sundress, and whether she preferred wine or beer with dinner. This contributed to a friendly atmosphere and made conversations easier. Crew members frequently asked the researcher what exactly she was doing. Once they understood that her focus was hand hygiene, they began to share many facts, feelings, and beliefs about it. They also felt they had found a competent person to answer their questions about germs, hygiene, pandemics, and related topics. The researcher experiences similar interactions during everyday life elsewhere, although in hospitals and universities (where she often organize hand hygiene trainings) staff members have different levels of knowledge about infection transmission.   Where are these results? Compliance data are presented on Figure 10. L721: “From the compliance data (Figure 10), we can see that…”   ******************  

 We appreciate the reviewers’ valuable feedback and the opportunity to improve our manuscript. We look forward to their evaluation of the revised version.
